# An update on multimodal imaging strategies for nipple discharge: from detection to decision

**DOI:** 10.1186/s13244-025-01947-1

**Published:** 2025-03-24

**Authors:** Mireia Pitarch, Rodrigo Alcantara, Laura Comerma, Ivonne Vázquez de Las Heras, Javier Azcona, Antonia Wiedemann, Maja Prutki, Eva Maria Fallenberg

**Affiliations:** 1 Department of Radiology and Nuclear Medicine, Hospital del Mar, Passeig Marítim de la Barceloneta, 25-29, 08003 Barcelona, Spain; 2 Department of Pathology, Hospital del Mar, Passeig Marítim de la Barceloneta, 25-29, 08003 Barcelona, Spain; 3 Department of Clinical Medicine, Institute of Diagnostic and Interventional Radiology, TUM School of Medicine & Health, Klinikum Rechts der Isar, Technical University of Munich, Munich (TUM), Ismaninger Str. 22, 81675 München, Germany; 4 Department of Radiology, Clinical Hospital Centre Zagreb, University of Zagreb School of Medicine, Kispaticeva 12, HR-10000 Zagreb, Croatia

**Keywords:** Breast, Nipple discharge, Diagnostic imaging

## Abstract

**Abstract:**

Nipple discharge affects over 80% of women at some point in their lives, with malignancy detected in up to 23% of cases. This review highlights the shift from traditional surgical approaches to advanced imaging techniques, which enhance diagnostic accuracy and reduce unnecessary procedures. Diagnosis begins with a thorough medical history and physical examination to assess the need for imaging. Physiological nipple discharge, which is bilateral, multiductal, and non-spontaneous, typically requires no imaging. Conversely, pathological nipple discharge (PND), characteristically unilateral, uniductal, and spontaneous, requires imaging to rule out malignancy. Bloody PND is frequently associated with breast cancer, and up to 12% of non-bloody PND cases also involve malignancy. For women over 40 years, the first-line imaging modality is full-field digital mammography (FFDM) or digital breast tomosynthesis (DBT), usually combined with ultrasound (US). Men with PND undergo FFDM/DBT starting at age 25 years due to their higher risk of breast cancer. For women aged 30–39 years, US is the first assessment tool, with FFDM/DBT added, if necessary, while US is preferred for younger women and men. When initial imaging is negative or inconclusive, magnetic resonance imaging (MRI) is useful, often replacing galactography. With its high sensitivity and negative predictive value of almost 100%, a negative MRI can often obviate the need for surgery. Contrast-enhanced mammography (CEM) offers a viable alternative when MRI is not feasible. Although invasive, ductoscopy helps identify patients who may not require duct excision. This review consolidates the latest evidence and proposes an updated diagnostic algorithm for managing PND effectively.

**Critical relevance statement:**

Effective management of nipple discharge requires recognising when imaging tests are needed and selecting the most appropriate diagnostic technique to rule out malignancy and avoid unnecessary interventions.

**Key Points:**

- First-line imaging for pathological nipple discharge (PND) assessment includes ultrasound and mammography.
- MRI is recommended for patients with PND and negative conventional imaging.
- A negative MRI is sufficient to justify surveillance rather than surgery.
- Contrast-enhanced mammography (CEM) is an alternative when MRI is unavailable or contraindicated.

**Graphical Abstract:**

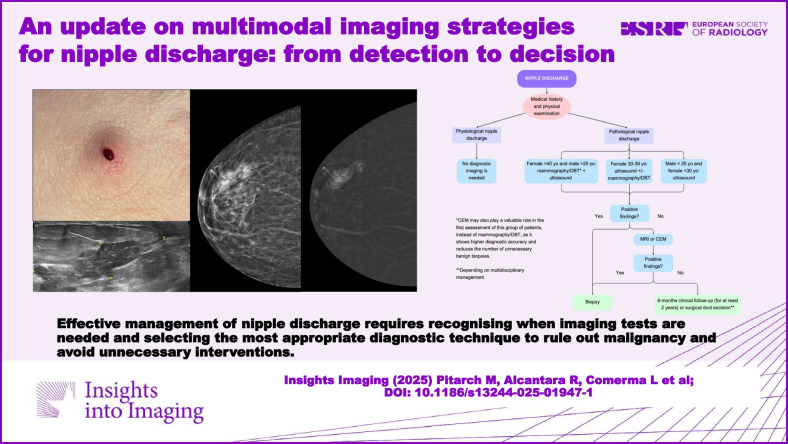

## Introduction

Nipple discharge is the third most common breast complaint after breast pain and palpable breast masses, with a prevalence ranging from 4.8% to 7.4% [1, 2]. More than 80% of women experience nipple discharge at some point in their lives [3].

Based on its characteristics and clinical context, nipple discharge can be classified as either physiological or pathological, with the latter requiring breast imaging assessment. Although most causes of pathological nipple discharge (PND) are benign, malignancy is detected in 3% to 23% of cases [4–9]. Consequently, the key challenge in managing this complaint is to exclude malignant lesions while minimising unnecessary surgical interventions.

This article reviews the diagnostic approach and management of nipple discharge in the current era of multimodal imaging and proposes a diagnostic algorithm. The article also discusses the main breast diseases causing PND, and their radiological and histological correlations.

## Clinical history, physical examination, and first laboratory tests

The first step in determining whether nipple discharge is physiological or pathological is a thorough medical history and physical examination. A detailed history helps to assess the patient’s health status, current medications, and risk factors for breast cancer, such as age and a personal and/or family history of the disease [10]. Physical examination includes inspecting and palpating both breasts and axillary regions. For non-spontaneous discharge, breast compression is required to assess the colour of the discharge and whether it is uniductal or multiductal. Clinical examination may also show other associated signs, such as palpable lumps, glandular asymmetry, or changes in the nipple-areolar complex. On examination, the presence of palpable suspicious lumps has also been related to a higher risk of breast cancer [11].

Physiological nipple discharge is usually bilateral, multiductal, and non-spontaneous. The discharge can be white, green, or yellow [3]. Lactational discharge, which occurs during pregnancy, lactation, or even after the first-year post-weaning, is considered normal and distinct from galactorrhoea [3, 12]. The most common cause of physiological nipple discharge is drug-induced hyperprolactinemia (particularly due to antipsychotics). When hyperprolactinemia is medication-related, discontinuing the drug, if possible, is recommended. Other causes include pregnancy, chronic renal failure, cirrhosis, pituitary adenomas, hypothalamic lesions, and unidentifiable causes [12–14].

PND is characteristically unilateral, uniductal, persistent and spontaneous, regardless of colour [15]. Nipple discharge showing any one of these characteristics can be regarded as pathological. The appearance is usually clear, serous, or bloody [3]. Malignancy is detected in up to 23% of all PND cases [6], and most patients with bloody PND are diagnosed with breast cancer [5, 16]. Some studies have found a significant association between bloody discharge and malignancy [17, 18]. However, the colour of the discharge alone cannot reliably indicate malignancy [19]. Clark et al [4] reported that a non-negligible percentage (9.5%–12.1%) of women with non-bloody nipple discharge had cancer, including 1 woman with green and another with milky discharge.

## Nipple discharge cytology

Nipple discharge cytology is performed by gently squeezing the areola and nipple to collect the discharge, which is then spread onto a glass slide. Although the procedure is simple, it may not yield results if there is no discharge during the examination.

Currently, there is no international classification system for nipple smear cytology, unlike breast fine-needle aspiration biopsy (FNAB) [20]. Patients with nipple discharge usually undergo a triple assessment consisting of clinical examination, breast imaging, and pathological analysis, often involving nipple discharge cytology [18, 21]. However, nipple smear cytology has limited diagnostic accuracy in ruling out malignancy [22], demonstrating poor sensitivity (11%–34.6%) but high specificity (81%–100%) in detecting malignancy in patients with nipple discharge [17, 18]. In addition, some studies report a false-negative rate of up to 65% [1, 17, 23]. As a result, some experts advise against the routine use of cytology in the workup of nipple discharge [24, 25].

## Imaging techniques

### Mammography

Diagnostic breast imaging is not necessary for physiological nipple discharge [4]. However, when PND is suspected, an initial imaging evaluation with FFDM and ultrasound (US) is normally carried out.

According to the American College of Radiology (ACR) Appropriateness Criteria for the evaluation of nipple discharge [24], the first-line imaging technique in women aged 40 years or older is either FFDM or DBT, usually complemented by US. For women aged 30 to 39 years, the ACR supports the use of either FFDM/DBT or US as the initial imaging modality, depending on institutional preference and individual patient factors. While the incidence of breast cancer before the age of 40 years is low—approximately 7% [26, 27]—some experts recommend US as the initial imaging assessment in this age group to minimise the cumulative risk of malignancy from ionising radiation. FFDM/DBT may be added only if risk factors for breast cancer are present or if malignancy is suspected based on US findings [28, 29]. Furthermore, in symptomatic women aged 30 to 39 years, US has higher sensitivity than FFDM (95.7% versus 60.9%) [30].

In contrast, due to the high incidence of breast cancer in men with PND, imaging assessment in those older than 25 years should start with FFDM/DBT, usually supplemented by US at the same appointment [24]. In women younger than 30 years and men younger than 25 years, the initial imaging technique is US [24].

**Fig. 1:**
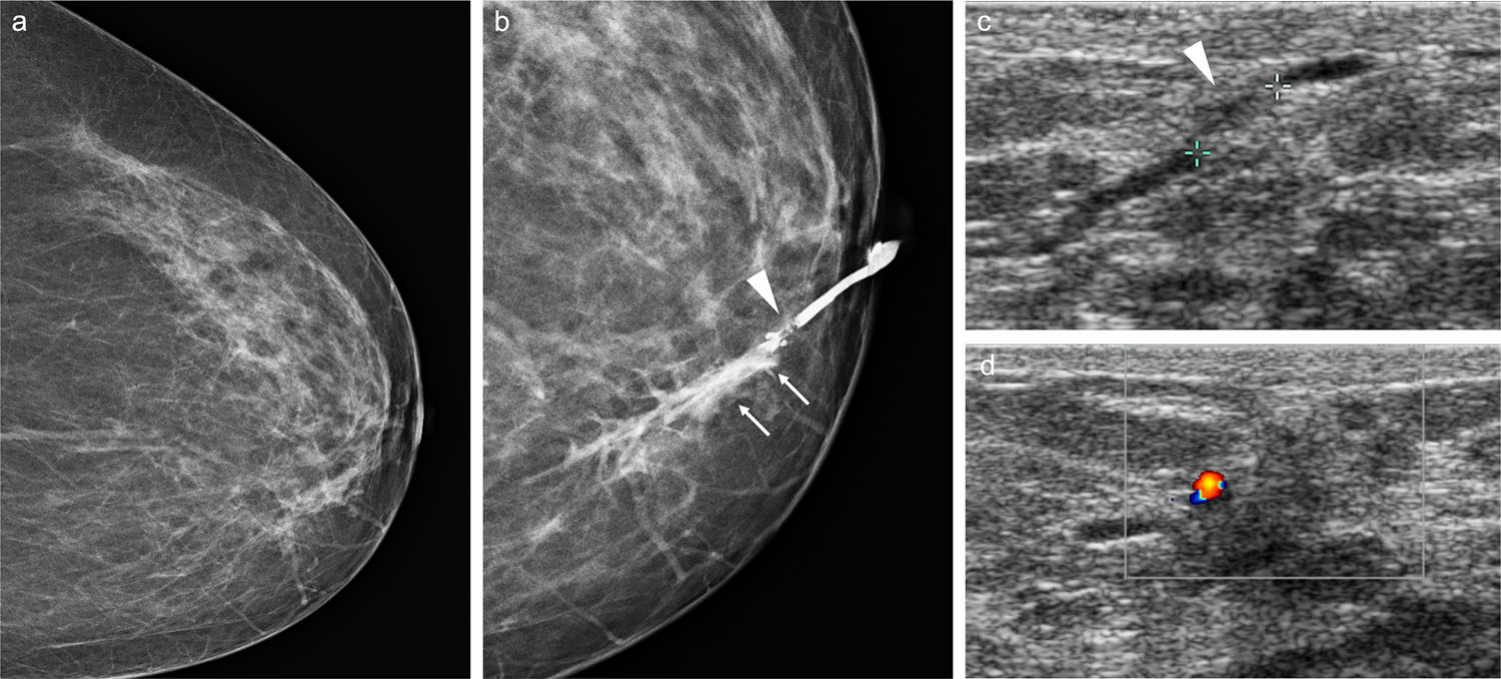
Intraductal papilloma. A 63-year-old woman with serous left PND. Cytology was negative. **a** Mammogram (craniocaudal view) showing no abnormalities. **b** Galactography revealing a ductal “cut-off” and a filling defect near the nipple (arrowhead), with periductal contrast extravasation in peripheral ducts (arrows). **c** Ultrasound depicting duct ectasia and a 5-mm intraductal mass (arrowhead), which exhibits colour Doppler signal (**d**). An ultrasound-guided core-needle biopsy (CNB) was performed. Histological analysis confirmed fragments of intraductal papilloma without atypia. The patient declined surgical excision and was subsequently lost to follow-up

The sensitivity of FFDM in this clinical context is low, ranging from 7% to 68% [6, 7, 31, 32], due to the small size of some lesions, their intraductal location, or the absence of calcifications [1] (Fig. 1). Moreover, the retroareolar region is highly complex anatomically due to the convergence of numerous structures. Therefore, additional mammographic views, such as magnification or spot-compression of the subareolar region, may enhance diagnostic accuracy.

### Digital breast tomosynthesis

DBT reduces the risk of missing lesions caused by overlapping breast tissues. A randomised, open-label, superiority trial [33] demonstrated that DBT combined with synthesised 2D mammography significantly increased the detection rate of invasive breast cancer compared to FFDM alone (7.1 versus 4.8 cases per 1000 women screened). Although DBT involves a slightly higher radiation dose than FFDM, it remains well within international safety standards and is considered acceptable [34].

Data on the use of DBT to assess nipple discharge are limited. A retrospective study [35] analysed the diagnostic performance of DBT compared with FFDM, US, and MRI in 53 patients with nipple discharge. Both MRI and DBT demonstrated 100% sensitivity, but DBT had higher specificity than MRI (82.9% versus 61.9%), suggesting its potential as an alternative to MRI in the diagnostic workup of patients with this condition. Further research is needed to corroborate these findings.

### Ultrasound

According to the European Society of Breast Imaging (EUSOBI) guidelines, US should be the first diagnostic approach for clinical abnormalities in women younger than 40 years [29]. If US reveals suspicious findings or there are personal and family risk factors for malignancy, FFDM or DBT is indicated.

The sensitivity and specificity of US in women with nipple discharge vary widely in the literature, ranging from 36% to 83% and 12% to 84%, respectively [7]. When combined with colour and power Doppler, this technique allows solid intraductal lesions to be differentiated from secretions, with some studies reporting 100% diagnostic accuracy [36].

Accurate assessment of the retroareolar region with US requires precise technique to avoid artefacts in the form of air bubbles between the probe, the skin, and the nipple [37]. The probe should be positioned perpendicularly to the major axis of the duct to better assess its entire length and delineate any intraductal lesions [38].

Contrast-enhanced ultrasonography (CEUS) has also been analysed in the assessment of intraductal lesions [39]. The reported data show that CEUS may be useful to differentiate benign from malignant lesions. A key parameter is the enlarged enhancement scope, defined as enhancement of the area of the lesion relative to the surrounding breast parenchyma. This enlargement, which is due to the infiltrative growth of malignant ductal lesions, has been identified as a useful independent risk factor for malignancy [39–42].

A prospective study of 82 suspicious ductal lesions [39] reported that calcifications were more frequently detected in malignant duct lesions than in benign lesions (42.1% versus 4.5%, *p* < 0.001) and identified them as an independent predictor of malignancy on US (OR = 8.96, *p* = 0.047). Significant associations have also been reported between calcifications visible on US and high-grade ductal carcinoma in situ (DCIS) [43, 44].

### Galactography

Galactography, also known as ductography, involves the mammographic visualisation of a pathological duct after the instillation of contrast medium. Nipple discharge must be present at the time of the procedure. Once a 30-gauge blunt cannula is inserted, a volume of 0.2–0.3 mL of non-ionic iodinated contrast medium is carefully administered [45]. Magnified craniocaudal and 90-degree lateral views of the subareolar region are then obtained [45–47], with additional views taken if needed. The aim of galactography is to identify ductal abnormalities, such as intraductal filling defects, duct wall irregularities, ductal obstruction, and ductal expansion [46, 48].

While galactography is commonly considered unable to discriminate between benign and malignant lesions [49], some studies indicate otherwise [8, 48]. A retrospective study [8] analysed the diagnostic performance of galactography and MRI in 146 patients with PND. They reported that galactography demonstrated a sensitivity of 77.4% and a specificity of 75.7% in detecting neoplastic and high-risk lesions, while these figures were 85.7% and 71.4% for MRI. The authors concluded that galactography remains a practical, valuable, and cost-effective diagnostic tool, which should not be routinely replaced by MRI. They further suggested MRI should be performed when galactography is technically unsuccessful [9].

Another imaging modality recently being investigated is the use of galactography with DBT, with studies comparing the use of DBT with FFDM or synthesised 2-dimensional mammography in association with galactography. Galactography using DBT has shown increased sensitivity, specificity, and diagnostic accuracy than full-field digital galactography [50, 51].

Galactography has long been the gold standard for assessing PND when FFDM and US findings are negative. However, as an invasive test that can cause discomfort and has several limitations—such as difficulty cannulating the duct, the risk of periductal contrast extravasation, and contrast-related adverse effects—it is increasingly considered obsolete in modern multimodal breast imaging. Consequently, breast MRI has replaced galactography in many centres [47, 52, 53].

### Ductoscopy

Ductoscopy consists of the real-time visualisation of the duct by inserting a 0.9-mm micro-endoscope into the affected duct [3]. Local anaesthesia is usually required to improve patient tolerance to the test.

The main aim of ductoscopy is to facilitate targeted surgical excision, avoiding the resection of healthy breast tissue. With advances in microendoscopic tools, ductoscopy not only allows direct visualisation of lesions but also the performance of intraductal breast biopsies, including endoscopic vacuum-assisted biopsy, and even lesion excision [54]. The therapeutic role of ductoscopy has mainly been investigated in patients with PND, and no suspicious findings on conventional imaging [54–57].

A recent meta-analysis comparing the diagnostic performance of MRI and ductoscopy in detecting malignancy in patients with PND [58] found that ductoscopy had significantly higher diagnostic accuracy (95.3%) than MRI (83.6%), suggesting its usefulness in identifying patients who could avoid duct excision surgery. Another meta-analysis [59] concluded that, due to the high sensitivity of ductoscopy and the low incidence of malignancy in PND, this technique offers a negative predictive value (NPV) of 98%–100%. Thus, normal findings on ductoscopy may indicate that surgery is unnecessary.

The main limitation of ductoscopy is the length and outer diameter of the probe, which prevents visualisation of the more distal branches. Other drawbacks are that it is invasive, expensive, and time-consuming [60].

### Magnetic resonance imaging

Both the ACR and the EUSOBI recommend contrast-enhanced MRI in patients with PND and negative findings on conventional imaging [24, 61]. The incidence of malignancy in these patients ranges from 5.7% to 17% [15, 49, 62, 63].

MRI is widely recognised as the most sensitive imaging modality for detecting breast cancer in all risk groups. Its overall sensitivity for malignancy is approximately 90% [61], with a range of 71% to 100% [64]. However, MRI may fail to detect lesions with low enhancement, such as low-grade DCIS or small invasive carcinomas [65–67].

The diagnostic performance of MRI in patients with PND and negative conventional imaging has been thoroughly investigated. One study [15] reported MRI to have a sensitivity of 85.71% and specificity of 98.53% in detecting malignancy, with a positive predictive value (PPV) of 92.31% and an NPV of 97.1%. Another study of 103 women [68] found 100% sensitivity but lower specificity (68%), with a PPV of 37% and an NPV of 100%. Similarly, a third study [62] showed that the risk of breast cancer after negative or inconclusive results on conventional imaging and a negative MRI was less than 4%.

Comparative studies have shown that the diagnostic accuracy of MRI is superior to that of galactography. A systematic review and meta-analysis [52] found that the sensitivity and specificity of MRI for causative lesions were significantly higher than those of galactography (92% versus 69% and 76% versus 39%, respectively). Another study [66] confirmed the superior sensitivity and specificity of MRI in detecting pathological findings (95.7% versus 85.7% and 69.7% versus 33.3%, respectively).

The high sensitivity and NPV of MRI suggest that a negative MRI result may eliminate the need for surgery in this symptomatic patient population [53, 68]. Unlike ductoscopy or galactography, breast MRI assesses not only the secretory duct but also the entire ductal system and surrounding breast tissue without requiring duct cannulation. Moreover, MRI effectively identifies causative lesions located far from the nipple-areolar complex and can rule out contralateral breast cancer [3, 69].

### Contrast-enhanced mammography

Another promising technique for assessing patients with PND is CEM. Unlike FFDM, CEM provides information on both anatomical and perfusion-related changes, regardless of breast density. Similar to MRI, CEM detects tumour neoangiogenesis using an intravenous contrast medium (in this case, an iodinated contrast agent). Recent meta-analyses [70, 71] have validated the diagnostic accuracy of CEM in women with clinical and/or radiological suspicion of breast cancer, reporting sensitivity ranging from 91% to 96% and specificity from 74% to 77%. These findings confirm CEM as a reliable alternative when FFDM and US yield inconclusive results or when MRI is unavailable or contraindicated.

**Fig. 2:**
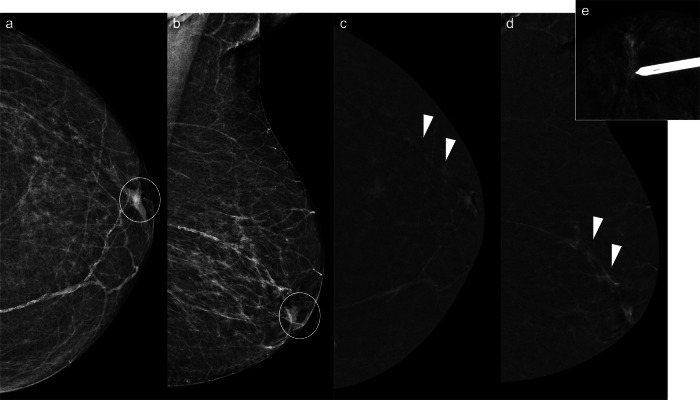
Contrast-enhanced mammography-guided biopsy. An 85-year-old woman with left bloody PND and nipple retraction. Cytology was negative. **a** Craniocaudal and (**b**) mediolateral oblique low-energy CEM images depicting nipple retraction (circle), with no other abnormalities. **c** Craniocaudal and (**d**) mediolateral oblique recombined CEM images revealing a linear, non-mass-like enhancement extending from the upper outer quadrant to the nipple (arrowheads). No lesions were detected on ultrasound (not shown). The patient underwent a (**e**) CEM-guided vacuum-assisted biopsy (VAB). Histological analysis confirmed intermediate-grade DCIS, ER++, PR−−, HER2−, Ki67 20%

With the recent introduction of CEM-guided biopsy, MRI guidance is no longer the only option for assessing enhancing-only lesions [72]. CEM-guided biopsy is a more widely available technique (Fig. 2) and has shown similar success and complication rates to MRI-guided biopsy [72].

CEM offers additional advantages over MRI, including being more cost-effective and quicker to perform, and is better tolerated by patients [73]. A prospective study of 207 patients [74] reported that CEM reduced the benign biopsy rate in screening recalls by 16.4%, suggesting that it could be a valuable one-stop morpho-functional method in breast units. The utility of routine use of US in patients with normal CEM results is controversial, as the US may lead to unnecessary benign biopsies [75, 76].

The main limitations of CEM are the risks associated with iodinated contrast agent reactions and contrast-induced nephropathy, although their reported incidence is less than 1% [77, 78]. In addition, CEM requires a higher radiation dose than FFDM, although it remains within the limits established by the Mammography Quality Standards Act [79, 80].

In the context of PND, CEM has been shown to outperform the combination of US and FFDM. A prospective study of 140 patients [81] demonstrated that CEM had superior sensitivity (97.5% versus 92.6%) and NPV (94.1% versus 84.2%) to US plus FFDM. CEM was also more effective in detecting multifocality, multicentricity, and diffuse abnormalities. In a recent study evaluating the feasibility of CEM in patients with bloody nipple discharge [82], CEM demonstrated higher sensitivity (100% versus 89.7%), specificity (62.1% versus 34.5%), and diagnostic accuracy (81.3% versus 62.1%) compared to combined FFDM and US in the detection of breast cancer. In that study, CEM was able to effectively differentiate benign from malignant lesions with significant predictors of malignancy, including enhancing lesions ≥ 1.5 cm (*p*-value 0.025) and suspicious morpho-functional features such as irregular shape, irregular/spiculated margins, and segmental/linear non-mass-like enhancements (NME) (*p*-value < 0.001). Although further studies are needed, these results suggest that CEM is a valuable diagnostic tool in women with PND that may modify patient management [83].

## Surgery

The main priority in the workup and management of nipple discharge is to identify underlying malignancy while avoiding unnecessary surgical interventions [68, 84].

Traditionally, patients with PND have undergone surgery for both diagnostic and therapeutic purposes [6, 85, 86]. Surgical options range from major duct excision to the resection of a single affected duct (microdochectomy), which aims to preserve the continuity between the nipple and the subareolar ductal system. However, surgery is associated with complications, such as an inability to breastfeed, retroareolar necrosis, and the possibility of missing peripheral lesions [3].

The reported malignancy rate in patients with PND undergoing duct excision surgery ranges from 9.3% to 37% [6, 85]. Higher rates have been attributed to the inclusion of patients with clinical abnormalities, such as a palpable lump or suspicious radiological findings, and those not undergoing US during the initial diagnostic workup [85]. Nevertheless, a systematic review [84] showed that the weighted mean malignancy rate after surgery in patients with negative findings on conventional imaging and no other clinical abnormalities was 8.1% (ranging from 2.3% to 13.5%) [84], indicating that approximately 9 out of 10 patients undergo surgery for benign conditions.

With the advent of MRI, which offers high sensitivity and NPV, surgery may be avoided in patients not requiring an intervention for symptomatic relief [47, 49, 53, 68]. CEM seems to be a good alternative to MRI, given its similar diagnostic accuracy, wider availability, lower cost, and better patient tolerance [73, 87]. When available, ductoscopy may also be useful in selecting patients who would not benefit from surgery.

The development of image-guided percutaneous biopsy techniques, especially vacuum-assisted biopsy, has enabled the percutaneous removal of many lesions causing PND, provided they are anatomically accessible (i.e., not too close to the nipple or skin). This approach has been increasingly used in benign lesions and is now employed in most high-risk lesions [88–90].

When underlying pathology has been ruled out, surgery may still be an option for symptomatic relief, especially if nipple discharge persists beyond a 2-year follow-up [3].

## Causes of pathological nipple discharge

### Benign causes

Nipple discharge is usually attributed to benign conditions, with the main causes being intraductal papilloma and duct ectasia. Other benign entities that may present as PND are periductal or plasma cell mastitis, fibrocystic changes, nipple adenoma, epidermal inclusion cysts in the areolar region, syringomatous tumours of the nipple, and retroareolar cysts in adolescents [37, 69, 91].

#### Intraductal papilloma

Papillary lesions are a heterogeneous group that includes both benign and malignant lesions. The most frequent cause of nipple discharge is intraductal papilloma (IP), accounting for 36.8% to 57% of cases [31, 92]. Pathologically, IP is a benign mass-like projection consisting of papillary fronds attached to the inner mammary duct wall by a fibrovascular core, lined by epithelial and myoepithelial cells [93, 94].

**Fig. 3:**
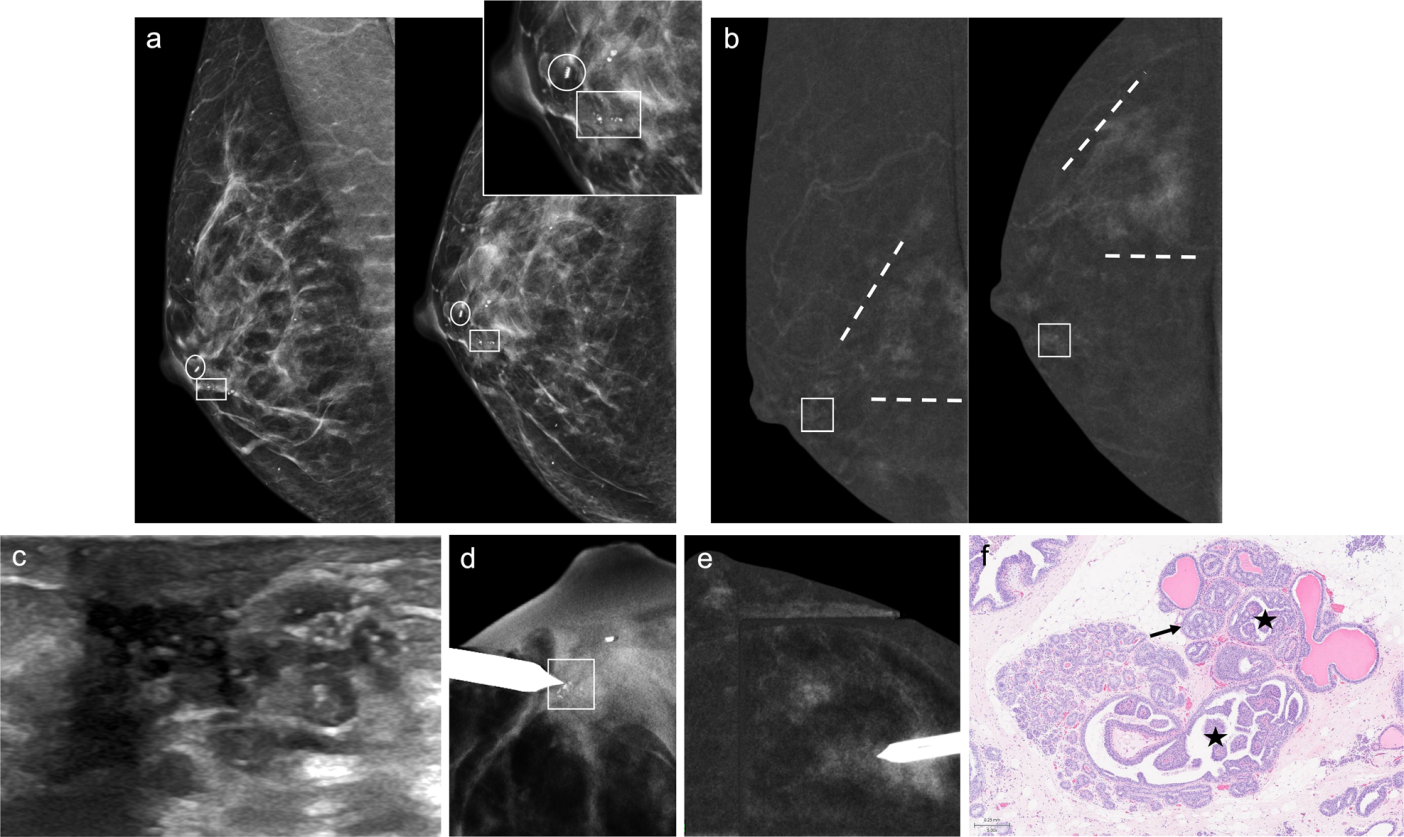
Multiple intraductal papillomas with malignant upgrade to DCIS. A 48-year-old woman with a personal history of right breast intraductal papilloma surgery and bloody PND in the same breast. Cytology was negative. **a** Synthesised 2-dimensional mammogram showing retroareolar coarse heterogeneous calcifications (square) and a clip from a prior benign biopsy (circle). **b** Recombined CEM images displaying differential contrast enhancement near the calcifications (square) and segmental non-mass-like enhancement (NME) in the outer quadrants (discontinuous lines). **c** Ultrasound showing a retroareolar solid intraductal lesion with calcifications; ultrasound-guided VAB was negative. **d** Tomosynthesis-guided VAB of the retroareolar calcifications, and (**e**) CEM-guided VAB of the NME showing multiple intraductal papillomas and atypical ductal hyperplasia in the latter. The patient underwent a right mastectomy. **f** Histology of the specimen showing several papillomas (stars), consisting of benign intraductal proliferations with branching fibrovascular cores lined by epithelial and myoepithelial cells, and DCIS (arrow), a neoplastic intraductal epithelial proliferation with cytological and architectural atypia (haematoxylin-eosin stain, 5x)

IP can be classified as solitary or multiple and as central or peripheral. Solitary papillomas are usually central, arising in the ducts in the retroareolar region. These tumours commonly affect perimenopausal women and are usually associated with nipple discharge [95, 96]. In contrast, peripheral papillomas are usually multiple (Fig. 3), arise in the terminal ductal lobular units, and occur in younger patients. Peripheral papillomas are more often associated with malignancy than solitary IP [96].

**Fig. 4:**
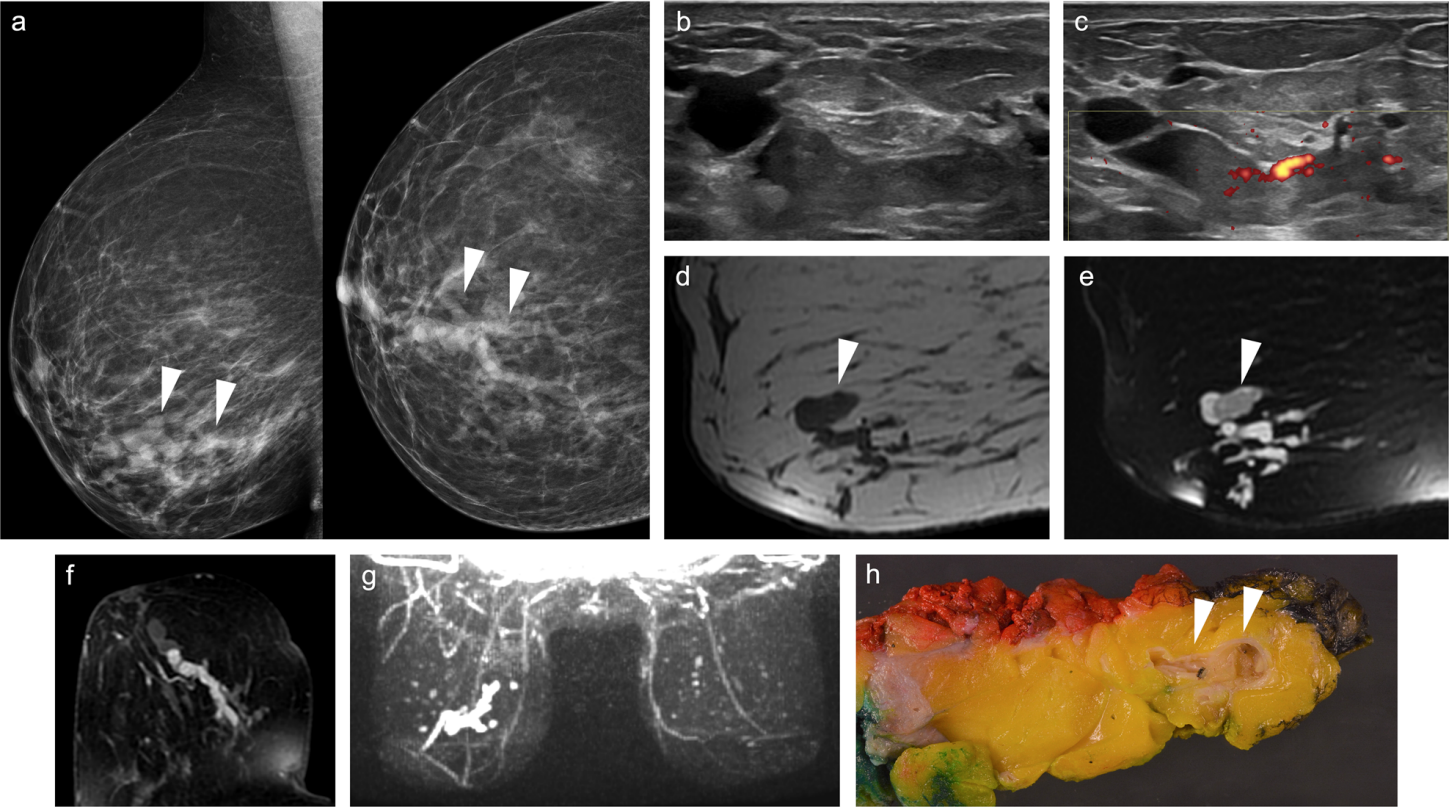
Intraductal papilloma with duct dilatation. A 50-year-old woman with serous right PND. Cytology was negative. **a** Mammogram showing tubular structures in the lower inner quadrant (arrowheads). **b** Ultrasound showing duct ectasia and a solid intraductal mass with colour Doppler signal (**c**). **d** Sagittal T1-weighted MRI showing hypointense duct ectasia, obscuring the intraductal mass (arrowhead). **e** Sagittal fat-saturated T2-weighted MRI showing hyperintense duct ectasia with a hypointense intraductal solid mass (arrowhead), corresponding to linear NME on (**f**) axial contrast-enhanced T1-weighted MRI and (**g**) 3-dimensional maximum-intensity projection (MIP) subtracted image. An ultrasound-guided CNB was performed. Histologic study confirmed a partially sclerosed intraductal papilloma with usual ductal hyperplasia. **h** Macroscopic photograph of the fresh specimen after surgical excision, inking, and sectioning showing the lesion (arrowheads)

Central IP is usually occult on mammography, unless the lesion is sufficiently large to be visualised or contains calcifications [97]. On US, it may appear as an intraductal mass, usually associated with duct dilatation (Fig. 4), an intracystic mass, or a solid mass with well-defined margins [98].

On MRI, intraductal lesions are hypointense on T2- and T1-weighted imaging and show enhancement on post-contrast dynamic T1-weighted sequences. In post-contrast sequences, multiple peripheral papillomas may show a linear or segmental NME that may resemble DCIS [99].

A retrospective study of 45 patients [100] found MRI to have significantly higher sensitivity than CEM in the diagnosis of IP (100% versus 65%, respectively) among lesions of all sizes, particularly in lesions smaller than 5 mm.

Fragments from a benign papilloma in a core-needle biopsy (CNB) are classified as lesions of uncertain malignant potential (B3 lesions), as IP can coexist with higher-grade malignant lesions [101, 102]. When IP with atypia, such as atypical ductal hyperplasia (ADH), is identified on CNB, the upgrade rate to DCIS or invasive carcinoma upon excision is significantly higher compared to IP without atypia (26.9% versus 2.3%, respectively) [90]. Vacuum-assisted biopsy (VAB) is the procedure of choice for suspected papillary lesions due to its superior diagnostic accuracy [88]. IP with ADH should be surgically excised. For IPs without atypia that are completely removed by VAB, imaging surveillance is sufficient [90]. However, for IPs without atypia that are either too large or anatomically inaccessible for percutaneous removal, open excision is recommended [88, 103]. Some studies suggest that active surveillance alone may be an appropriate alternative to surgery for benign papillomas with radiologic-pathologic concordance to avoid overtreatment [104–106]. Multidisciplinary teams should guide therapeutic decisions for each B3 lesion.

#### Duct ectasia

Duct ectasia is the second most common cause of PND, accounting for 5.5% to 33% of cases [31, 107]. This benign process is characterised by dilatation of the mammary ducts and is thought to result from periductal inflammation and fibrosis [108]. Duct ectasia usually affects the ducts of the subareolar region symmetrically and bilaterally and is more frequent in patients older than 50 years [107]. While often asymptomatic, duct ectasia may present as nipple discharge, palpable subareolar mass, or nipple retraction [109].

On mammography, duct ectasia appears as radiodense tubular or branching structures converging at the nipple. Rod-like calcifications radiating towards the nipple may also be visualised due to calcified intraductal inspissated contents [110].

On US, benign duct ectasia may appear as tubular structures that can be anechoic or contain echoes due to debris or infection. If an intraductal mass is suspected, which typically exhibits internal vascularity on Doppler US, histological confirmation is warranted [37]. Sonographic features that suggest a malignant process include the peripheral location of the duct ectasia, wall irregularity, focal thickening of the duct wall, and the presence of surrounding hypoechogenicity. These features should arouse suspicion, and a biopsy should be considered to rule out malignancy [111, 112].

**Fig. 5:**
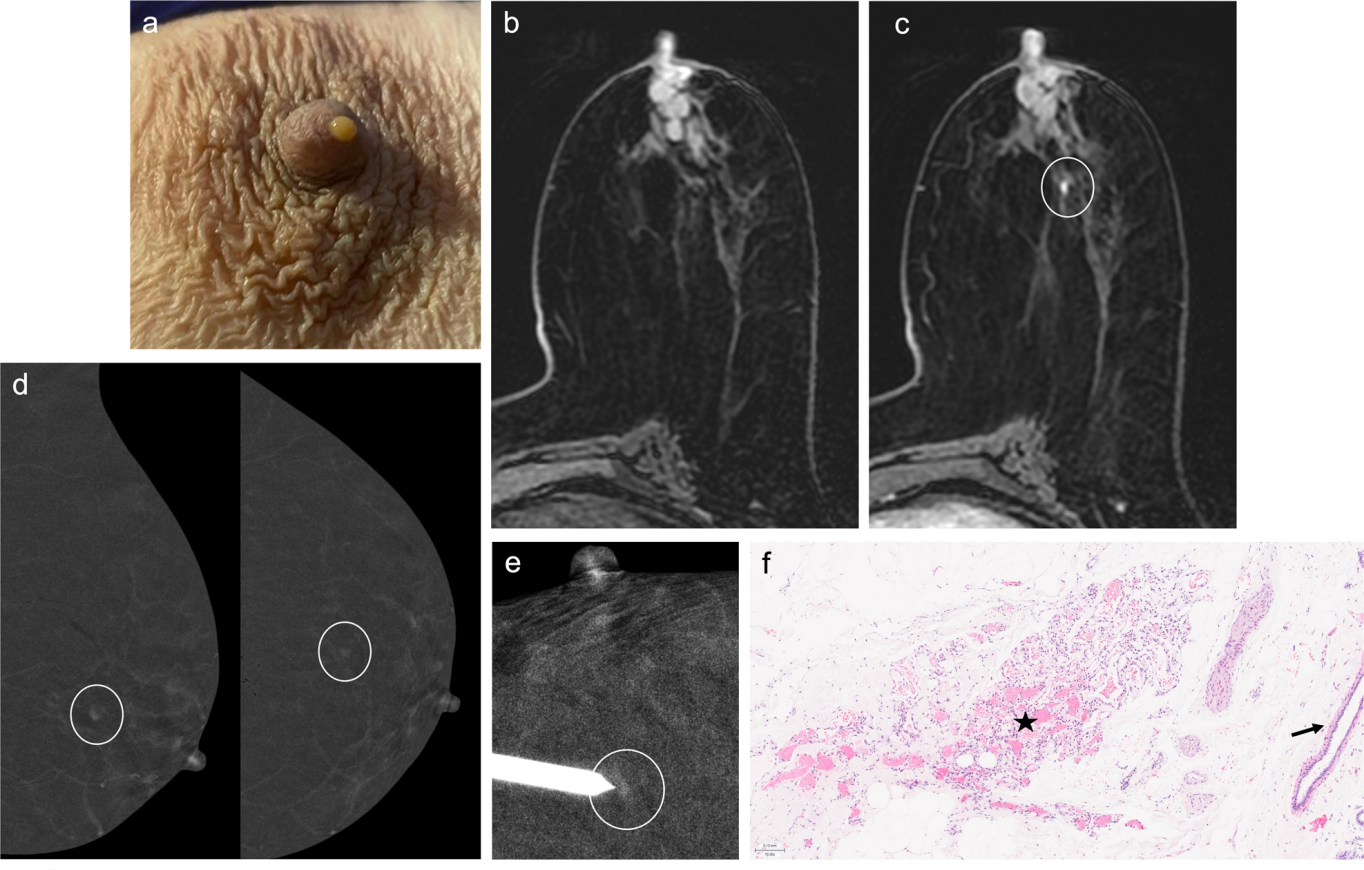
High-Signal Intensity Ducts on T1-Weighted MRI. A 73-year-old woman with sero-brownish left PND. Cytology was negative. **a** Photograph of the discharge. Mammography and ultrasound showed no abnormalities (not shown). **b** Axial fat-suppressed pre-contrast T1-weighted MRI showing retroareolar hyperintense duct ectasia. **c** Axial fat-suppressed post-contrast T1-weighted MRI revealing a 4-mm focus in the retroareolar region (circle), perfectly correlating with (**d**) recombined CEM images (circle). **e** A CEM-guided VAB was conducted. **f** Biopsy showing a 3.5-mm capillary haemangioma (star) in the breast parenchyma. Note the presence of a breast duct adjacent to the haemangioma (arrow) (haematoxylin-eosin stain, 10x)

On MRI, duct ectasia usually appears as tubular structures converging at the nipple-areolar complex, with high-signal intensity on T2-weighted imaging and variable signal intensity on T1-weighted imaging. Proteinaceous debris or blood shows high-signal intensity relative to parenchyma on pre-contrast T1-weighted sequences (Fig. 5). While duct ectasia does not usually show enhancement, it may exhibit a pattern of enhancement that mimics DCIS when associated with inflammation (i.e., rounded, smooth-margined ring enhancement, or even heterogeneous NME) [37, 113].

A solitary dilated duct, appearing as an asymmetric tubular or branching structure in the retroareolar region, may also present as PND [114]. The Breast Imaging Reporting and Data System (BI-RADS) 5th edition recommends further imaging and biopsy for solitary dilated ducts identified on mammography, if no benign cause is evident [115]. However, recent data confirm that a solitary dilated duct is a rare condition seldom related to malignancy, with malignancy rates reported to range from 0% to 3.4% [114, 116, 117]. When asymptomatic and not accompanied by suspicious findings, such as an associated mass, internal vascularity, architectural distortion, or calcifications, these ducts may be considered benign [114, 116, 118]. Additional US evaluation is key to identifying any associated suspicious imaging features [118].

#### Periductal mastitis or plasma cell mastitis

Another benign cause of PND is periductal mastitis (PM), which is a chronic inflammatory condition of the breast characterised by dilation of the subareolar mammary ducts, infiltration of plasma cells, and eventual abscess formation [119, 120]. This condition mainly affects non-lactating premenopausal women [121]. Independent risk factors for PM include obesity and the late onset of menarche [119]. While the association with smoking is controversial [119], tobacco has been related to recurrent episodes of PM [122].

PM may present as breast pain, a palpable mass, nipple discharge, nipple retraction, or breast abscess, which can lead to mammary duct fistula [120]. The mammographic appearance of PM is non-specific and may range from being undetectable to showing retroareolar focal asymmetry or thickening of the areolar skin [120, 123–125]. Rod-like calcifications may also be associated with this condition [125].

**Fig. 6:**
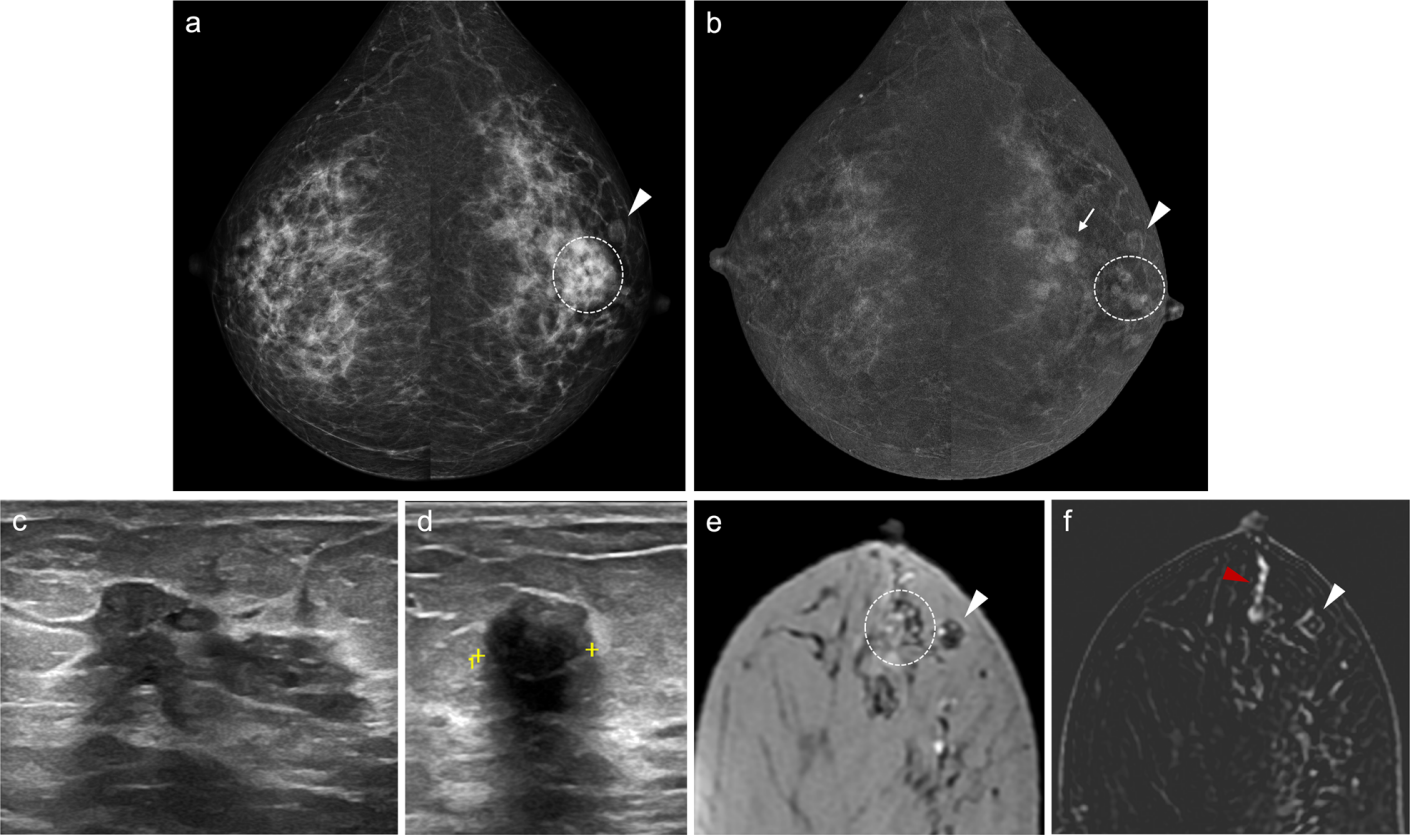
Periductal mastitis mimicking breast cancer. A 49-year-old-woman with bloody left PND. **a** Craniocaudal low-energy CEM images depicting a left retroareolar asymmetry (discontinuous circle) and a circumscribed mass in the retroareolar region of the outer quadrant (arrowhead), both with mild heterogeneous enhancement on (**b**) recombined CEM image. The arrow points to a histologically proven fibroadenoma. Ultrasound revealed retroareolar duct ectasia with debris (**c**) and a slightly irregular hypoechoic nodule with posterior acoustic shadowing (**d**). An ultrasound-guided biopsy of the mass histologically confirmed duct ectasia with dense fibrosis and periductal inflammatory infiltrates of plasma cells and macrophages (periductal mastitis). **e** Axial T2-weighted MRI showing hyperintense retroareolar dilated ducts (discontinuous circle) and the biopsied mass in the outer quadrants (arrowhead), which on (**f**) axial post-contrast T1-weighted MRI (subtracted image) display no suspicious enhancement (white arrowhead). Instead, a single enhancing duct with wall thickening is depicted (red arrowhead). The pathological duct was excised, revealing duct ectasia with periductal inflammation and hyperplasia

On US, PM may appear as dilated mammary ducts with inspissated secretions and increased periductal colour Doppler signal, related to periductal inflammation [37]. It can also present as an irregular, heterogeneous, hypoechoic mass that may mimic breast carcinoma [120, 123] (Fig. 6). MRI shows a wide spectrum of features, including non-mass-like enhancing lesions, ring-like enhancements, and irregular enhancing masses.

PM is normally treated with antibiotics and drainage if there is abscess formation. If this treatment is ineffective, a biopsy is recommended [123]. The overall recurrence rate of PM is 50% [124]. Therefore, surgery is recommended for recurrence, as it reduces the recurrence rate by up to 28% [126]. Mammary duct irrigation with corticosteroids and antimicrobial agents has also shown good therapeutic efficacy in PM [124, 127].

#### Nipple adenoma

**Fig. 7:**
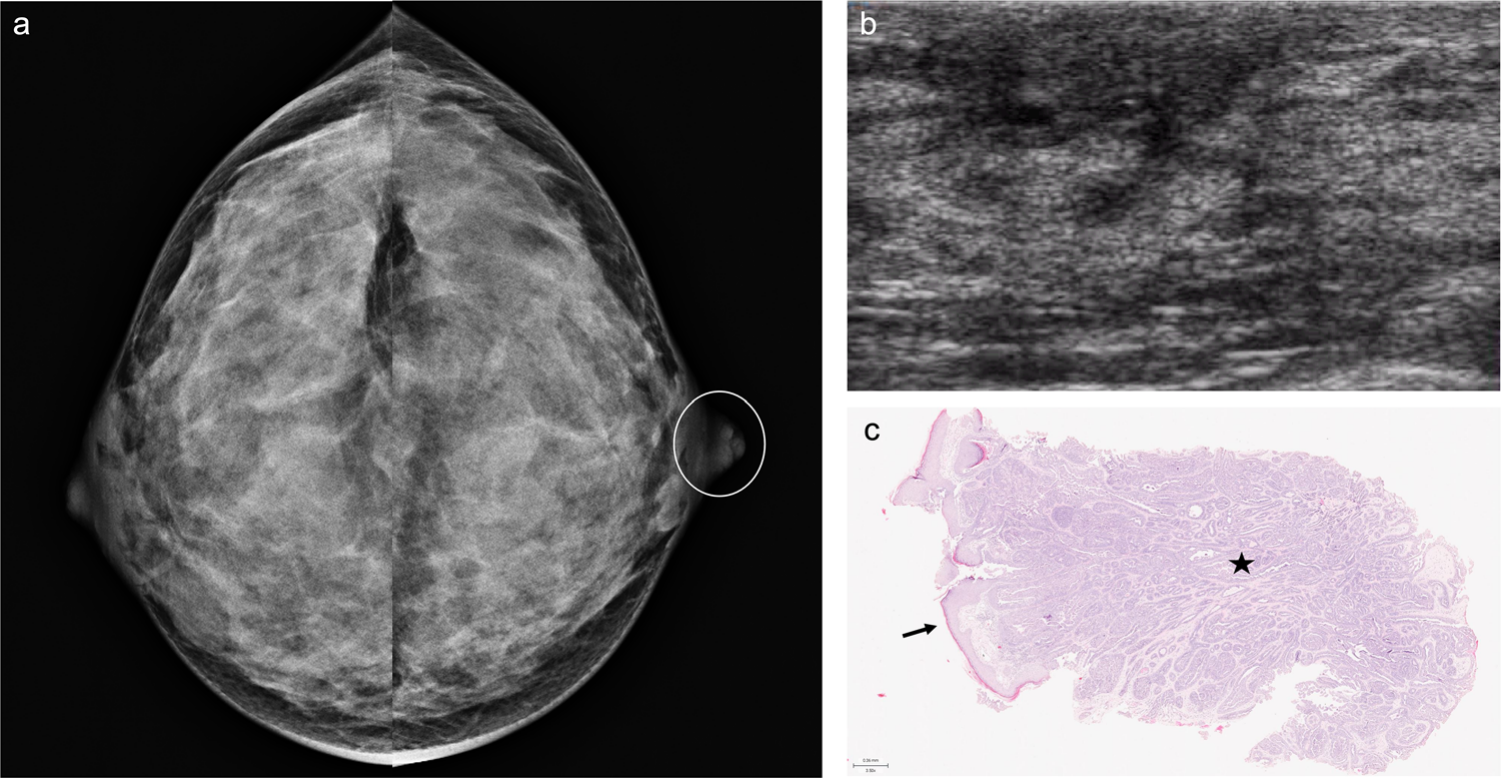
Nipple adenoma. A 33-year-old woman with bloody left PND, accompanied by tenderness, redness, and swelling of the left nipple. **a** Mammogram showing enlargement and asymmetry of the left nipple. **b** Ultrasound depicting no focal lesions within the nipple. **c** Punch biopsy confirmed an intact epidermis (arrow) with the underlying benign proliferation of compact glandular structures with epithelial hyperplasia and a retained myoepithelial cell layer (star), suggestive of nipple adenoma (haematoxylin-eosin stain, 3.5x)

Nipple adenoma, also known as florid papillomatosis, erosive adenomatosis, or subareolar papillomatosis, is a benign glandular proliferation of the superficial lactiferous ducts within the nipple [128] that commonly affects middle-aged women. This benign nipple lesion presents as erosion and discharge in up to 83% of patients [129], often mimicking Paget disease [128]. Therefore, histological examination through skin punch biopsy is required for diagnosis. Mammographically, nipple adenoma may go unnoticed or manifest as a superficial mass within the nipple or local thickening of the nipple-areolar complex (Fig. 7) [129]. On US, it may appear as a homogeneous hypoechoic mass within the nipple that typically shows hypervascularity and may be associated with duct dilatation [130]. Surgical removal is recommended.

### Malignant causes

Malignancy associated with nipple discharge is mainly due to DCIS, which accounts for up to 86% of cases [131]. Most patients with breast carcinoma who present with PND have early-stage cancer, usually DCIS or stage I disease [132, 133]. Paget disease associated with DCIS may also present as nipple discharge [91].

**Fig. 8:**
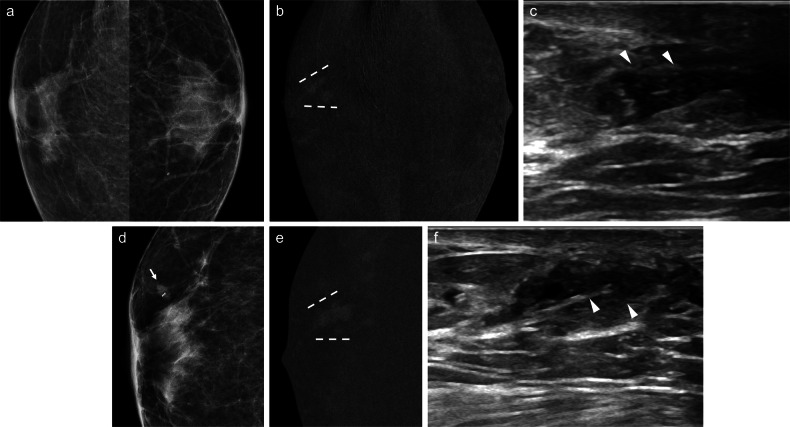
Breast cancer in a man with nipple discharge. A 73-year-old man with gynaecomastia and right bloody PND. **a** Craniocaudal low-energy CEM images showing diffuse bilateral gynaecomastia. **b** Craniocaudal recombined CEM images revealing a non-mass-like enhancement distributed segmentally in the retroareolar region of the right breast (discontinuous lines). **c** Ultrasound identifying an intraductal solid mass (arrowheads). Initial ultrasound-guided biopsy showed a fibroepithelial lesion without atypia, leading to surveillance. One year later, persistent discharge prompted revaluation. **d** Right craniocaudal low-energy CEM image showing a displaced post-biopsy clip (arrow). **e** Recombined CEM image showing a slight enlargement of the pathological enhancement (discontinuous lines). **f** Ultrasound revealing duct wall irregularity associated with the intraductal lesion (arrowheads). An ultrasound-guided VAB confirmed malignancy. The patient underwent mastectomy and sentinel lymph node biopsy (SLNB). Final histological analysis revealed invasive ductal carcinoma + DCIS + encapsulated papillary carcinoma. ER 3+, PR 3+, her2−, Ki67 10%. SLNB was negative

Although nipple discharge is uncommon in men, it is associated with breast cancer in 23%–57% of cases [134, 135]. Therefore, nipple discharge in men should always be considered a suspicious finding (Fig. 8).

#### Ductal carcinoma in situ

Up to 12% of patients with DCIS have nipple discharge [136]. Blood-stained nipple discharge increases the risk of DCIS and invasive ductal carcinoma [137]. The most common presentation of DCIS is asymptomatic calcifications, found in approximately 80% of cases [138]. Since DCIS is the most common malignancy associated with PND, FFDM should be thoroughly examined for any suspicious calcifications [47]. Fine pleomorphic, fine-linear, or fine-linear branching calcifications are usually associated with high-grade DCIS or DCIS with necrosis, whereas low and intermediate-grade DCIS are found in round, amorphous, or coarse heterogeneous morphology [136, 138].

**Fig. 9:**
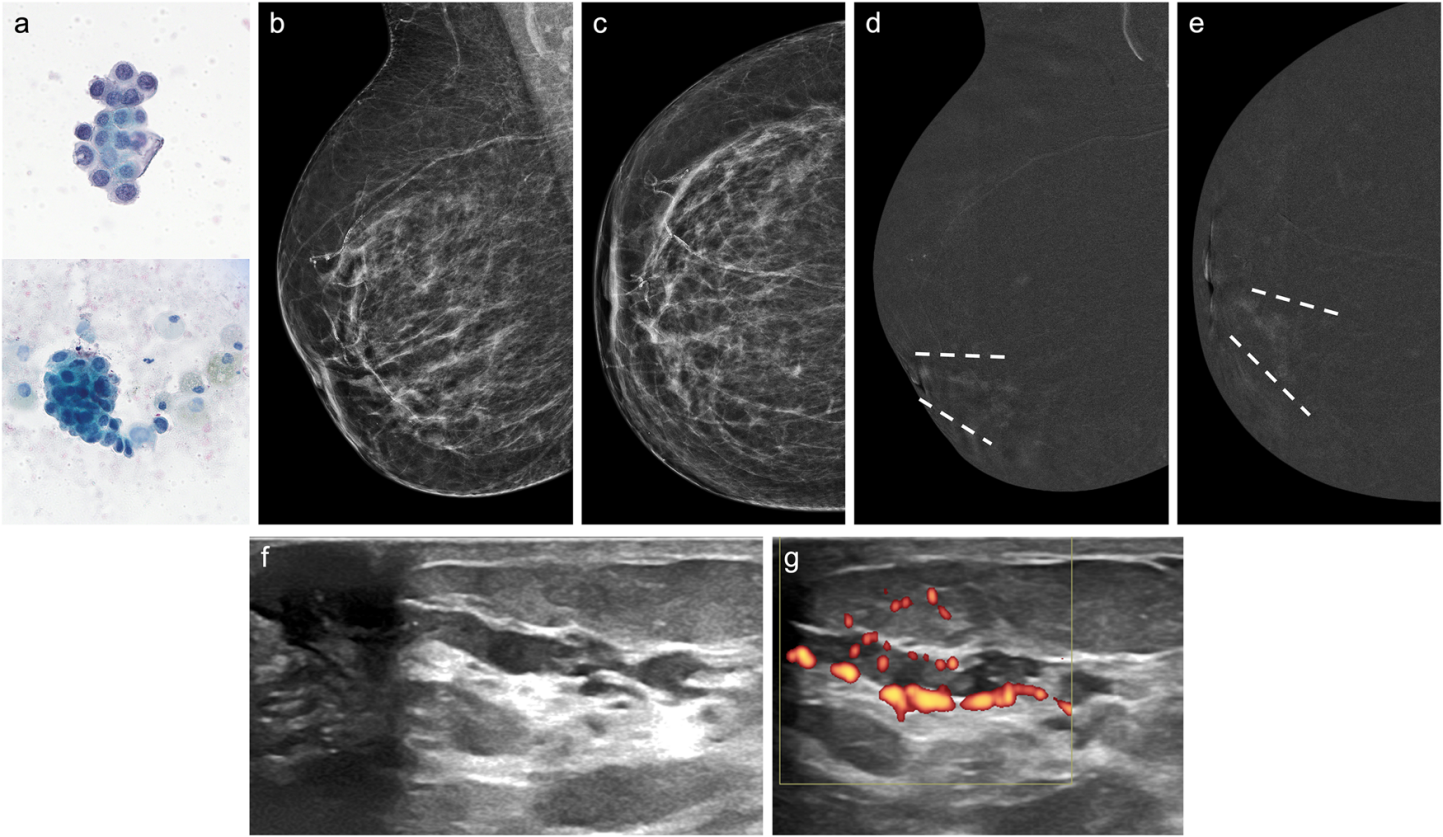
Non-calcified DCIS. A 78-year-old woman with right bloody PND. **a** Cytology showing abundant atypical epithelial cells arranged in plaques and groups with a papillary appearance (Papanicolaou stain, 60x). **b** Mediolateral oblique and (**c**) craniocaudal low-energy CEM images show no abnormalities. **d** Mediolateral oblique and, (**e**) craniocaudal recombined CEM images showing a segmental NME in the lower inner quadrant (discontinuous lines). **f** Ultrasound depicting duct ectasia with solid content, exhibiting colour Doppler signal (**g**). An ultrasound-guided CNB was performed. Histological analysis confirmed intermediate-grade DCIS, ER 3+, PR 3+, her2−, Ki67 20%

Nipple discharge is one of the most common clinical presentations of non-calcified DCIS (Fig. 9), along with palpable mass [139]. However, FFDM has a false-negative rate of up to 49% in the detection of non-calcified DCIS [140]. In this context, CEM offers an advantage, as it depicts both calcified and non-calcified DCIS, and is highly useful in defining the extent of the disease. Unlike MRI, CEM is also effective in identifying calcifications [141]. US can detect approximately 50% of mammographically visible calcified DCIS [138], with even higher detection rates for non-calcified DCIS (95%) compared with FFDM (68%) or DBT (84%) [142]. On US, non-calcified DCIS may appear as ductal abnormalities, masses with mixed solid and cystic components, or areas of architectural distortion, which may resemble invasive carcinoma [139]. On MRI, the most common presentation of DCIS is clumped NME with linear or segmental distribution [123, 139]. Similarly, DCIS is also usually detected as NME on CEM [143].

#### Invasive carcinoma

Invasive carcinoma—including ductal, lobular, papillary, or mucinous subtypes—can also present as PND, predominantly in association with DCIS [15, 96, 144, 145]. Although rare, invasive carcinoma without an in situ component may also occasionally present as PND [131, 133, 137]. However, it is the in situ component, rather than the invasive component, that is typically associated with PND.

Invasive carcinoma presenting as PND does not exhibit distinct imaging features beyond those characteristic of its histological subtype. A retrospective study of 60 patients [133] found that most patients with breast carcinoma and PND (76.7%) reported nipple discharge as their only symptom, while the remainder had a palpable breast mass. Among those with PND alone, DCIS was the most common histology (56.5%), whereas invasive ductal carcinoma with DCIS was most frequent in patients with both PND and a breast mass (57.1%).

**Fig. 10:**
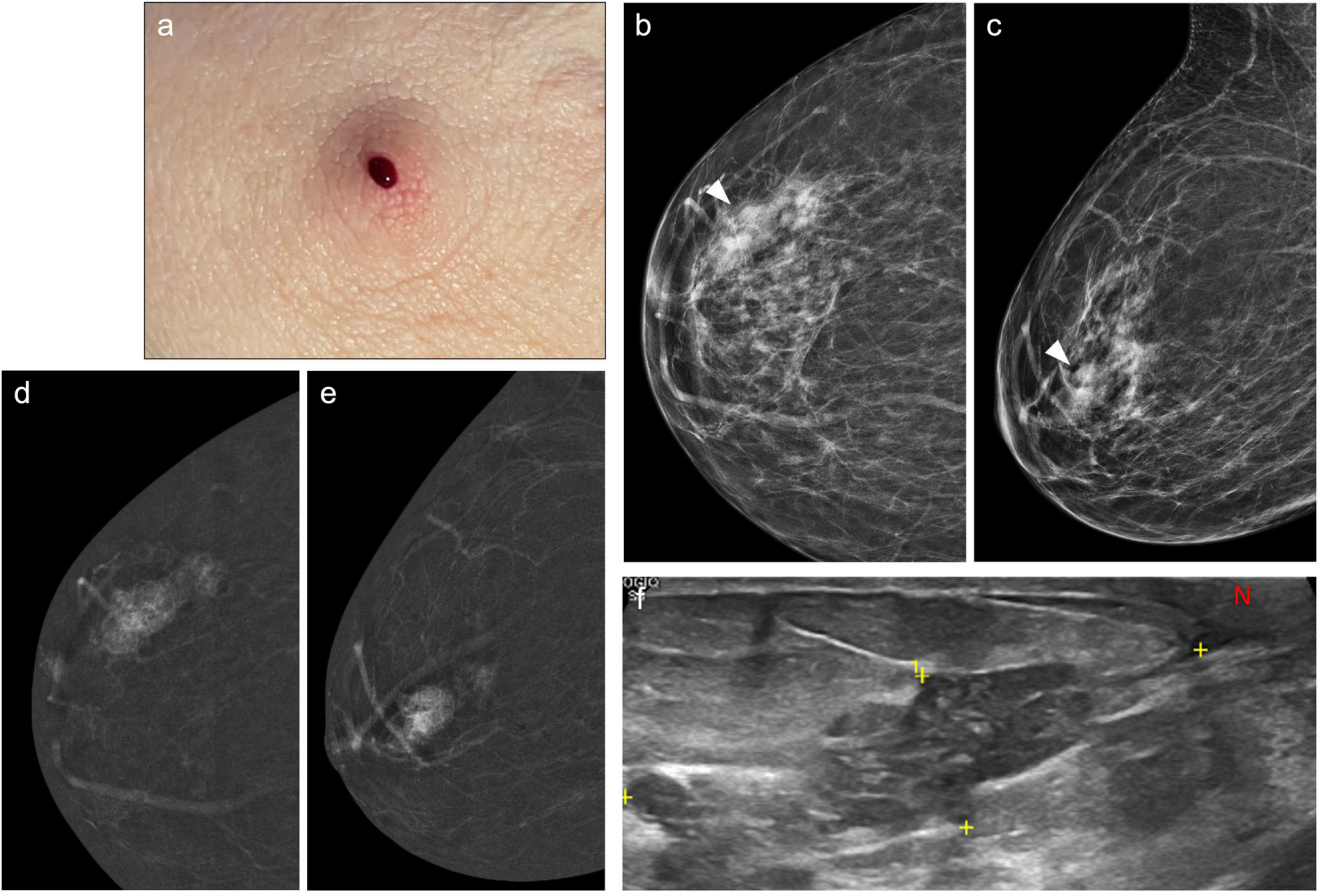
Invasive and in situ carcinoma. A 71-year-old woman with bloody PND from the right breast. **a** Photograph of the discharge. **b** Craniocaudal and (**c**) mediolateral oblique low-energy CEM images depicting a dense mass with obscured margins in the junction between the outer quadrants (arrowhead); **d** On craniocaudal and (**e**) mediolateral oblique recombined CEM images, the lesion shows heterogeneous enhancement. **f** Ultrasound scan revealing a hypoechoic irregular mass (delimited by yellow pointers) in contact with a subareolar duct (*N* = nipple). An ultrasound-guided core-needle biopsy was performed. Histological analysis confirmed a papillary lesion with marked atypia and areas of DCIS, invasive ductal carcinoma, invasive lobular carcinoma, intermediate-grade ER 3+, PR 3+, HER2−, Ki67 30%

On FFDM, invasive breast cancers often appear as focal asymmetries or masses with variable margin characteristics (Fig. 10), sometimes associated with calcifications [146]. On US, the most common features related to invasive cancers are an irregular mass, non-parallel orientation, and hypoechoic or complex echogenicity [96, 147]. On MRI, the appearance of invasive carcinomas varies depending on the histological subtype.

## Nipple discharge diagnostic algorithm

**Fig. 11:**
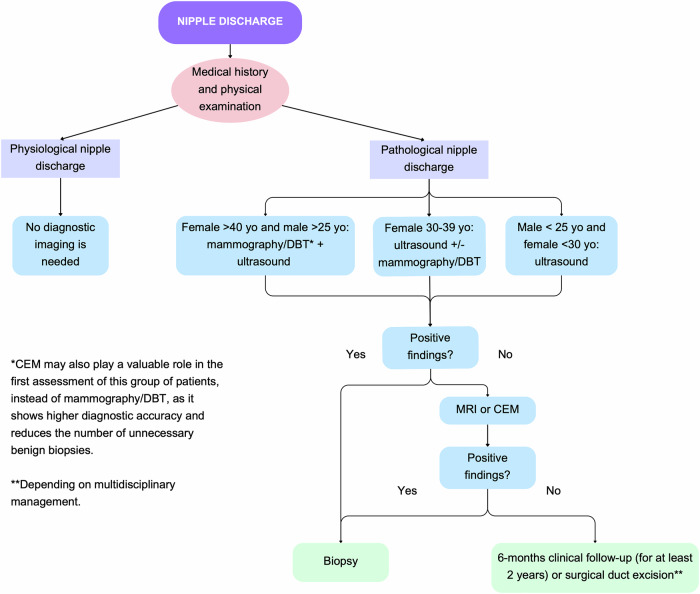
Diagnostic algorithm for nipple discharge

The first steps in assessing nipple discharge are a medical history and physical examination. In physiological nipple discharge (i.e., bilateral, multiductal, and non-spontaneous), breast imaging is unnecessary. However, for PND (i.e., unilateral, uniductal, and spontaneous), an initial imaging evaluation with FFDM and/or US is recommended, depending on the patient’s age and gender. For men aged > 25 years and women aged > 40 years, FFDM or DBT is the first-line imaging technique, usually complemented by US. For women aged 30 to 39 years, the initial imaging assessment is US, with the addition of FFDM/DBT if there are associated risk factors or findings suggesting malignancy on US. For men < 25 years and women < 30 years, the initial recommended technique is US. If the findings of conventional imaging are negative or inconclusive, further evaluation with MRI is recommended, while CEM is a valid method when MRI is contraindicated or unavailable. CEM may also play a useful role in the initial assessment of patients with PND, especially in men > 25 years and women > 40 years (instead of a FFDM/DBT), as it offers higher diagnostic accuracy and reduces the number of unnecessary benign biopsies [75, 76]. Galactography is recommended as a second-line alternative option to MRI. The diagnostic algorithm is illustrated in Fig. 11.

## Conclusions

Nipple discharge is a fairly common complaint in breast units. Cytology is not routinely recommended due to its high false-negative rate. When the discharge is unilateral, uniductal, persistent, and spontaneous, this condition is classified as PND, and breast imaging is mandatory to rule out malignancy. Although PND is usually caused by benign conditions, malignancy is detected in 3% to 23% of cases [4–9].

The first-line imaging techniques are US or FFDM/DBT combined with US, depending on the patient’s age and gender. If these modalities yield negative findings, MRI is the preferred next step over galactography. Due to its high sensitivity and NPV, a negative MRI result is sufficient to justify surveillance rather than surgery. CEM can be a suitable alternative when MRI is contraindicated or unavailable. Ductoscopy is also a promising tool to identify patients who may benefit from surgery.

## Data Availability

Data sharing is not applicable to this article as no datasets were generated or analysed during the study.

## Abbreviations

ACR: American College of Radiology
CEM: Contrast-enhanced mammography
CEUS: Contrast-enhanced ultrasonography
CNB: Core-needle biopsy
DBT: Digital breast tomosynthesis
DCIS: Ductal carcinoma in situ
EUSOBI: European Society of Breast Imaging
FFDM: Full-field digital mammography
IP: Intraductal papilloma
MRI: Magnetic resonance imaging
NME: Non-mass-like enhancement
NPV: negative predictive value
PM: Periductal mastitis
PND: Pathological nipple discharge
PPV: Positive predictive value
US: Ultrasound
